# Supplementary material for: Simulation and Machine Learning Assessment of P-Glycoprotein Pharmacology in the Blood–Brain Barrier: Inhibition and Substrate Transport
Source: Int J Mol Sci. 2025 Sep 17;26(18):9050. doi: 10.3390/ijms26189050 (PMC12469845; doi:10.3390/ijms26189050)
Supplement: Supplementary file 1 [file ijms-26-09050-s001.zip › ijms-3859506-supplementary.pdf]

Simulation and Machine Learning Assessment of P-Glycoprotein Pharmacology in the Blood–Brain Barrier: Inhibition and Substrate Transport

Christian Jorgensen [1,2,*], Elizabeth Oliphant [1], Milly Barker [1], Eduardo López Martínez [3], Saaihasamreen Thulasi [1], Holly Prior [1], Ben William Franey [1], Charley Gregory [1], Jerry Oluwasegun [1], Anjalee Ajay [1] and Roger R. Draheim [1]

1 School of Medicine, Pharmacy and Biomedical Sciences, Faculty of Science & Health, University of Portsmouth, Portsmouth PO1 2DT, UK; up2115139@myport.ac.uk (E.O.); milly.barker@myport.ac.uk (M.B.); saaihasamreen.thulasi@myport.ac.uk (S.T.); up2102739@myport.ac.uk (H.P.); up2096876@myport.ac.uk (B.W.F.); up2047688@myport.ac.uk (C.G.); jerry.oluwasegun@myport.ac.uk (J.O.); anjalee.ajay@myport.ac.uk (A.A.); roger.draheim@port.ac.uk (R.R.D.)
2 Department of Chemistry, Aarhus University, Langelandsgade 140, 8000 Aarhus C, Denmark
3 Laboratory of Computational Biophysics of Macromolecules, School of Chemical Sciences, Meritorious Autonomous University of Puebla (BUAP), Puebla 72570, Mexico; eduardo.lopezmar@alumno.buap.mx
* Correspondence: christian.jorgensen@port.ac.uk

Table of Contents

1. **List of Tables**

Table S1. Summary of the built homology models for this work. Mouse model templates were used based on the PDB id 4Q9K [1], 6UJN [2] and 7OTG [3]. All constructs had sequence similarity surpassing 85%, which make them suitable candidates.
Table S2. Quality assessment of the built homology models for this work. Mouse model templates were used based on the PDB id 4Q9K [1], 6UJN [2] and 7OTG [3]. All constructs had sequence similarity surpassing 85%, which make them suitable candidates.

Table S3. Centre-of-mass (COM) inhibitor-inhibitor distances as defined in Figure S3. Inhibition of P-gp by a dimer of tariquidar (PDB id 7A6E; cryo-EM, 3.60 Å) [4]. Inhibition of P-gp by a trimer of elacridar (PDB id 8Y6I; cryo-EM, 2.54 Å) [5].

Table S4. Centre-of-mass (COM) inhibitor distance to COM of apex (F335, F336). Inhibition of P-gp by a monomer of QZ-Leu, dimer of tariquidar and trimer of elacridar. Distances (*d*) from each monomer COM to the COM of residues 335 and 336 were averaged per ligand to reflect their positions within the multimeric complex. The SD (σ) was determined by calculating distances from each monomer to the individual COMs of residues 335 and 336, then sum their respective SDs to estimate variation around the original combined COM distance.

Table S5. Predicted IC_{50} and affinity probability obtained from Boltz-2 for the inhibitor QZ-X (X=Leu, Phe). Summary of the confidence metrics for structural prediction. The predicted Template Modeling (pTM; 0.7-1: good; 0.5-0.7: average; 0-0.5: low) score considers the overall fold accuracy of a single protein chain [27]. The Predicted inter-chain TM (ipTM; 0.8-1: good; 0.6-0.8: average; 0-0.6: low) score assesses the inter-chain interactions in multimeric models [27]. The Confidence Score (0.75-1: good; 0.5-0.75: average; 0-0.5 low) showcases the overall model quality. The Average predicted Local Distance Difference Test (pLDDT; 0.7-1: good; 0.5-0.7: average; 0-0.5: low) is the per-residue confidence metric [27], and reflects the local structural reliability. The Affinity Probability (0.75-1: good; 0.5-.075 average;

0-0.5: low) estimates the likelihood that a predicted protein-ligand complex corresponds to a true, energetically favorable binding interaction [27]. The IC_{50} denotes the half-maximal inhibitory concentration, and is defined as the modulator concentration required to inhibit 50% of target activity, with a lower value denoting a higher modulator potency. The predicted negative log_{10} of IC_{50} (pIC_{50}) correlates with the potency of inhibition, with a higher value predicting a greater inhibitory effect.

2. List of Figures

Figure S1. (a) Inhibition of P-gp by a monomer of QZ-Leu (PDB id 4Q9K; X-ray, 3.80 Å) [1], with the binding pose shown in van der Waals representation and associated chemical structure depicted. (b) Inhibition of P-gp by a dimer of tariquidar (PDB id 7A6E; cryo-EM, 3.60 Å) [4]. (c) Inhibition of P-gp by a trimer of elacridar (PDB id 8Y6I; cryo-EM, 2.54 Å) [5]. Here, ABC represents the central binding cavity of P-gp where Point A denotes residues 335 and 336, point B denotes E184 and point C denotes G827, all represented in VDW drawing style and color red and diffuse material. Inhibitors are represented in licorice drawing styles and color purple, yellow and orange for QZ-Leu, Tariquidar and Elacridar respectively.

Figure S2. (a) Inhibition of P-gp by a monomer of QZ-Val (PDB id 4Q9J; X-ray, 3.80 Å) [1] and QZ-Leu (PDB id 4Q9K; X-ray, 3.80 Å) [1], with the binding pose shown in van der Waals representation and associated chemical structure depicted. (b) Inhibition of P-gp by a dimer of tariquidar (PDB id 7A6E; cryo-EM, 3.60 Å) [4]. (c) Inhibition of P-gp by a trimer of elacridar (PDB id 8Y6I; cryo-EM, 2.54 Å) [5]. Here, ABC represents the central binding cavity of P-gp where Point A denotes residues 335 and 336, point B denotes E184 and point C denotes G827, all represented in VDW drawing style and color red and diffuse material. Inhibitors are represented in licorice drawing styles and color purple, yellow and orange for QZ-Leu, Tariquidar and Elacridar respectively.

3. List of Schemes

Scheme S1. Alignment of target human P-gp sequence (Uniprot accession number P08183) to template murine sequence.

1. List of Tables

Table S1. Summary of the built homology models for this work. Mouse model templates were used based on the PDB id 4Q9K [1], 6UJN [2] and 7OTG [3]. All constructs had sequence similarity surpassing 85%, which make them suitable candidates.

Template PDB id	Template organism	Target sequence (Uniprot ID)	Sequence identity (%)	Template reference	Template resolution
4q9k	Mouse	P08183	88.94	[1]	3.80 Å
6ujn	Mouse	P08183	88.75	[2]	3.98 Å
7otg	Mouse	P08183	89.23	[3]	5.40 Å

Table S2. Quality assessment of the built homology models for this work. Mouse model templates were used based on the PDB id 4Q9K [1], 6UJN [2] and 7OTG [3]. All constructs had sequence similarity surpassing 85%, which make them suitable candidates.

Template	Sequence identity (%)	Oligo-state	QSQE	Found by	Range	Coverage	Description
6UJN.1.A	88.75	Monomer	0.00	HHblits	31 - 2177	0.92	Polypeptide
7OTG.1.A	89.23	Monomer	0.00	HHblits	34 - 1275	0.92	Polypeptide
4Q9K.1.A	88.94	Monomer	0.00	HHblits	31 - 1279	0.93	Polypeptide

Table S3. Centre-of-mass (COM) inhibitor-inhibitor distances as defined in Figure S3. Inhibition of P-gp by a dimer of tariquidar (PDB id 7A6E; cryo-EM, 3.60 Å) [4]. Inhibition of P-gp by a trimer of elacridar (PDB id 8Y6I; cryo-EM, 2.54 Å) [5].

	7A6E: Tariquidar	8Y6I: Elacridar
1-2	9.75	11.2
1-3	-	17.9
2-3	-	9.62

Table S4. Centre-of-mass (COM) inhibitor distance to COM of apex (F335, F336). Inhibition of P-gp by a monomer of QZ-Leu, dimer of tariquidar and trimer of elacridar. Distances (d) from each monomer COM to the COM of residues 335 and 336 were averaged per ligand to reflect their positions within the multimeric complex. The SD (σ) was determined by calculating distances from each monomer to the individual COMs of residues 335 and 336, then sum their respective SDs to estimate variation around the original combined COM distance.

Inhibitor	$d \pm \sigma$ (Å)
4Q9K: QZ-Leu	10.3 ± 1
7A6E: Tariquidar	13.2 ± 4
8Y6I: Elacridar	16.5 ± 7

Table S5. Predicted IC_{50} and affinity probability obtained from Boltz-2 for the inhibitor QZ-X (X=Leu, Phe). Summary of the confidence metrics for structural prediction. The predicted Template Modeling (pTM; 0.7-1: good; 0.5-0.7: average; 0-0.5: low) score considers the overall fold accuracy of a single protein chain [27]. The Predicted inter-chain TM (ipTM; 0.8-1: good; 0.6-0.8: average; 0-0.6: low) score assesses the inter-chain interactions in multimeric models [27]. The Confidence Score (0.75-1: good; 0.5-0.75: average; 0-0.5 low) showcases the overall model quality. The Average predicted Local Distance Difference Test (pLDDT; 0.7-1: good; 0.5-0.7: average; 0-0.5: low) is the per-residue confidence metric [27], and reflects the local structural reliability. The Affinity Probability (0.75-1: good; 0.5-.075 average; 0-0.5: low) estimates the likelihood that a predicted protein-ligand complex corresponds to a true, energetically favorable binding interaction [27]. The IC_{50} denotes the half-maximal inhibitory concentration, and is defined as the modulator concentration required to inhibit 50% of target activity, with a lower value denoting a higher modulator potency. The predicted negative $\log_{10}$ of IC_{50} (pIC_{50}) correlates with the potency of inhibition, with a higher value predicting a greater inhibitory effect.

	Inhibitors	
	QZ-Leu	*QZ-Phe*
Predicted TM-Score (pTM)	0.763	0.770
Interface Predicted TM-Score (ipTM)	0.890	0.907
Confidence Score	0.743	0.736
Average predicted local distance difference test (pLDDT)	0.706	0.693
Affinity Probability	0.402	0.489
Predicted pIC_{50}	6.565	6.579
Predicted IC_{50} (nM)	272.3	263.6

2. List of Figures

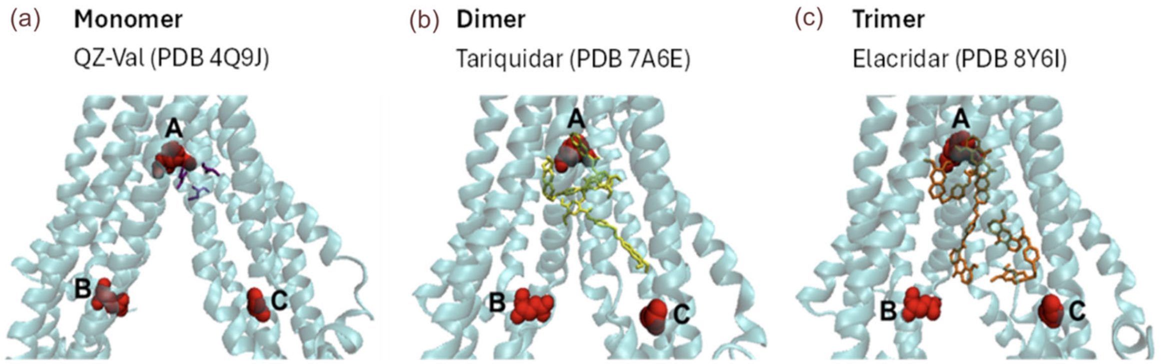

Figure S1. **Inhibition of P-gp by a monomer of QZ-Leu, a dimer of tariquidar and a trimer of elacridar**. (a) Inhibition of P-gp by a monomer of QZ-Leu (PDB id 4Q9K; X-ray, 3.80 Å) [1], with the binding pose shown in van der Waals representation and associated chemical structure depicted. (b) Inhibition of P-gp by a dimer of tariquidar (PDB id 7A6E; cryo-EM, 3.60 Å) [4]. (c) Inhibition of P-gp by a trimer of elacridar (PDB id 8Y6I; cryo-EM, 2.54 Å) [5]. Here, ABC represents the central binding cavity of P-gp where Point A denotes residues 335 and 336, point B denotes E184 and point C denotes G827, all represented in VDW drawing style and color red and diffuse material. Inhibitors are represented in licorice drawing styles and color purple, yellow and orange for QZ-Leu, Tariquidar and Elacridar respectively.

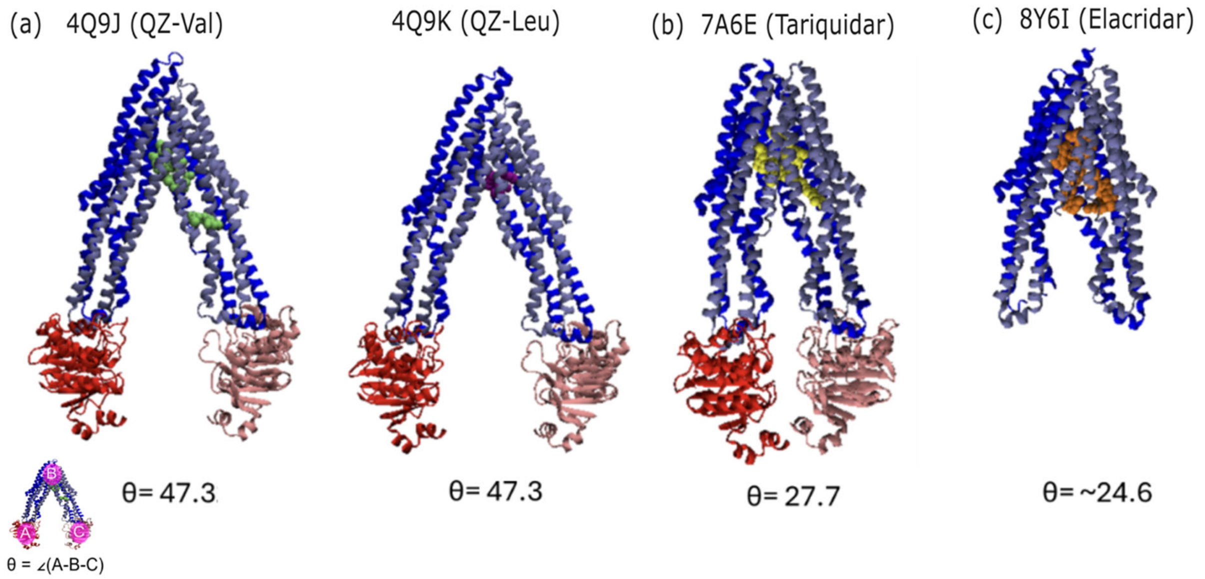

Figure S2. **Visualizing P-gp structures with bound inhibitors and their respective hinge angle θ.** (a) Inhibition of P-gp by a monomer of QZ-Val (PDB id 4Q9J; X-ray, 3.80 Å) [1] and QZ-Leu (PDB id 4Q9K; X-ray, 3.80 Å) [1], with the binding pose shown in van der Waals representation and associated chemical structure depicted. (b) Inhibition of P-gp by a dimer of tariquidar (PDB id 7A6E; cryo-EM, 3.60 Å) [4]. (c) Inhibition of P-gp by a trimer of elacridar (PDB id 8Y6I; cryo-EM, 2.54 Å) [5]. Here, ABC represents the central binding cavity of P-gp where Point A denotes residues 335 and 336, point B denotes E184 and point C denotes G827, all represented in VDW drawing style and color red and diffuse material. The angle θ is defined as the angle between the NBDs and the apex, with NBD1 denoted point A (residue 378-626), apex denoted point B (residue 335, 336), and NBD2 denoted point C (residue 1021 to 1272).

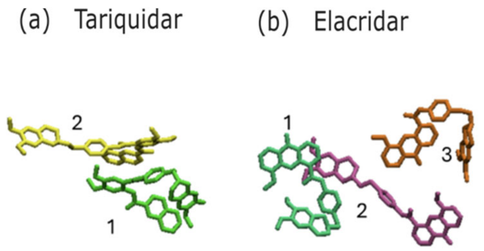

Figure S3. Internal geometry of dimeric and trimeric P-gp inhibitor. (a) Inhibition of P-gp by a dimer of tariquidar (PDB id 7A6E; cryo-EM, 3.60 Å) [4]. (b) Inhibition of P-gp by a trimer of elacridar (PDB id 8Y6I; cryo-EM, 2.54 Å) [5]. Inhibitors are represented in licorice drawing styles.

3. List of Schemes

Scheme S1. Alignment of target human P-gp sequence (Uniprot accession number P08183) [6] to template murine sequence.

```
CLUSTAL O(1.2.4) multiple sequence alignment
sp|P21447|MDR1A_MOUSE    MELEEDLKG-RADKNFSKMGKKSKKEKKEKKPAVSVLTMFRYAGWLDRLYMLVGTLAAII    59
sp|P08183|MDR1_HUMAN     MDLEGDRNGGAKKKNFFKLNNKSEKDKKEKKPTVSVFSMFRYSNWLDKLYMVVGTLAAII    60

sp|P21447|MDR1A_MOUSE    HGVALPLMMLIFGDMTDSFASVGNVSKN---STNMSEADKRAMFAKLEEEMTTYAYYYTG    116
sp|P08183|MDR1_HUMAN     HGAGLPLMMLVFGEMTDIFANAGNLEDLMSNITNRSDINDTGFFMNLEEDMTRYAYYYSG    120

sp|P21447|MDR1A_MOUSE    IGAGVLIVAYIQVSFWCLAAGRQIHKIRQKFFHAIMNQEIGWFDVHDVGELNTRLTDDVS    176
sp|P08183|MDR1_HUMAN     IGAGVLVAAYIQVSFWCLAAGRQIHKIRKQFFHAIMRQEIGWFDVHDVGELNTRLTDDVS    180

sp|P21447|MDR1A_MOUSE    KINEGIGDKIGMFFQAMATFFGGFIIGFTRGWKLTLVILAISPVLGLSAGIWAKILSSFT    236
sp|P08183|MDR1_HUMAN     KINEGIGDKIGMFFQSMATFFTGFIVGFTRGWKLTLVILAISPVLGLSAAVWAKILSSFT    240

sp|P21447|MDR1A_MOUSE    DKELHAYAKAGAVAEEVLAAIRTVIAFGGQKKELERYNNNLEEAKRLGIKKAITANISMG    296
sp|P08183|MDR1_HUMAN     DKELLAYAKAGAVAEEVLAAIRTVIAFGGQKKELERYNKNLEEAKRIGIKKAITANISIG    300

sp|P21447|MDR1A_MOUSE    AAFLLIYASYALAFWYGTSLVISKEYSIGQVLTVFFSVLIGAFSVGQASPNIEAFANARG    356
sp|P08183|MDR1_HUMAN     AAFLLIYASYALAFWYGTTLVLSGEYSIGQVLTVFFSVLIGAFSVGQASPSIEAFANARG    360

sp|P21447|MDR1A_MOUSE    AAYEVFKIIDNKPSIDSFSKSGHKPDNIQGNLEFKNIHFSYPSRKEVQILKGLNLKVKSG    416
sp|P08183|MDR1_HUMAN     AAYEIFKIIDNKPSIDSYSKSGHKPDNIKGNLEFRNVHFSYPSRKEVKILKGLNLKVQSG    420

sp|P21447|MDR1A_MOUSE    QTVALVGNSGCGKSTTVQLMQRLYDPLDGMVSIDGQDIRTINVRYLREIIGVVSQEPVLF    476
sp|P08183|MDR1_HUMAN     QTVALVGNSGCGKSTTVQLMQRLYDPTEGMVSVDGQDIRTINVRFLREIIGVVSQEPVLF    480

sp|P21447|MDR1A_MOUSE    ATTIAENIRYGREDVTMDEIEKAVKEANAYDFIMKLPHQFDTLVGERGAQLSGGQKQRIA    536
sp|P08183|MDR1_HUMAN     ATTIAENIRYGRENVTMDEIEKAVKEANAYDFIMKLPHKFDTLVGERGAQLSGGQKQRIA    540

sp|P21447|MDR1A_MOUSE    IARALVRNPKILLLDEATSALDTESEAVVQAALDKAREGRTTIVIAHRLSTVRNADVIAG    596
sp|P08183|MDR1_HUMAN     IARALVRNPKILLLDEATSALDTESEAVVQVALDKARKGRTTIVIAHRLSTVRNADVIAG    600

sp|P21447|MDR1A_MOUSE    FDGGVIVEQGNHDELMREKGIYFKLVMTQTAGNEIELGNEACKSKDEIDNLDMSSKDSGS    656
sp|P08183|MDR1_HUMAN     FDDGVIVEKGNHDELMKEKGIYFKLVTMQTAGNEVELENAADESKSEIDALEMSSNDSRS    660

sp|P21447|MDR1A_MOUSE    SLIRRRSTRKSICGPHDQDRKLSTKEALDEDVPPASFWRILKLNSTEWPYFVVGIFCAII    716
sp|P08183|MDR1_HUMAN     SLIRKRSTRRSVRGSQAQDRKLSTKEALDESIPPVSFWRIMKLNLTEWPYFVVGVFCAII    720

sp|P21447|MDR1A_MOUSE    NGGLQPAFSVIFSKVVGVFTNGGPPETQRQNSNLFSLLFLILGIISFITFFLQGFTFGKA    776
sp|P08183|MDR1_HUMAN     NGGLQPAFAIIFSKIIGVFTRIDDPETKRQNSNLFSLLFLALGIISFITFFLQGFTFGKA    780

sp|P21447|MDR1A_MOUSE    GEILTKRLRYMVFKSMLRQDVSWFDDPKNTTGALTTRLANDAAQVKGATGSRLAVIFQNI    836
sp|P08183|MDR1_HUMAN     GEILTKRLRYMVFRSMLRQDVSWFDDPKNTTGALTTRLANDAAQVKGAIGSRLAVITQNI    840

sp|P21447|MDR1A_MOUSE    ANLGTGIIISLIYGWQLTLLLLAIVPIIAIAGVVEMKMLSGQALKDKKELEGSGKIATEA    896
sp|P08183|MDR1_HUMAN     ANLGTGIIISFIYGWQLTLLLLAIVPIIAIAGVVEMKMLSGQALKDKKELEGSGKIATEA    900

sp|P21447|MDR1A_MOUSE    IENFRTVVSLTREQKFETMYAQSLQIPYRNAMKKAHVFGITFSFTQAMMYFSYAACFRFG    956
sp|P08183|MDR1_HUMAN     IENFRTVVSLTQEQKFEHMYAQSLQVPYRNSLRKAHIFGITFSFTQAMMYFSYAGCFRFG    960

sp|P21447|MDR1A_MOUSE    AYLVTQQLMTFENVLLVFSAIVFGAMAVGQVSSFAPDYAKATVSASHIIRIIEKTPEIDS    1016
sp|P08183|MDR1_HUMAN     AYLVAHKLMSFEDVLLVFSAVVFGAMAVGQVSSFAPDYAKAKISAAHIIMIIEKTPLIDS    1020

sp|P21447|MDR1A_MOUSE    YSTQGLKPNMLEGNVQFSGVVFNYPTRPSIPVLQGLSLEVKKGQTLALVGSSGCGKSTVV    1076
sp|P08183|MDR1_HUMAN     YSTEGLMPNTLEGNVTFGEVVFNYPTRPDIPVLQGLSLEVKKGQTLALVGSSGCGKSTVV    1080

sp|P21447|MDR1A_MOUSE    QLLERFYDPMAGSVFLDGKEIKQLNVQWLRAQLGIVSQEPILFDCSIAENIAYGDNSRVV    1136
sp|P08183|MDR1_HUMAN     QLLERFYDPLAGKVLLDGKEIKRLNVQWLRAHLGIVSQEPILFDCSIAENIAYGDNSRVV    1140

sp|P21447|MDR1A_MOUSE    SYEEIVRAAKEANIHQFIDSLPDKYNTRVGDKGTQLSGGQKQRIAIARALVRQPHILLLD    1196
sp|P08183|MDR1_HUMAN     SQEEIVRAAKEANIHAFIESLPNKYSTKVGDKGTQLSGGQKQRIAIARALVRQPHILLLD    1200

sp|P21447|MDR1A_MOUSE    EATSALDTESEKVVQEALDKAREGRTCIVIAHRLSTIQNADLIVVIQNGKVKEHGTHQQL    1256
sp|P08183|MDR1_HUMAN     EATSALDTESEKVVQEALDKAREGRTCIVIAHRLSTIQNADLIVVFQNGRVKEHGTHQQL    1260
```

sp|P21447|MDR1A_MOUSE	LAQKGIYFSMVSVQAGAKRS	1276
sp|P08183|MDR1_HUMAN	LAQKGIYFSMVSVQAGTKRQ	1280

References

1. Szewczyk, P.; Tao, H.; McGrath, A.P.; Villaluz, M.; Rees, S.D.; Lee, S.C.; Doshi, R.; Urbatsch, I.L.; Zhang, Q.; Chang, G. Snapshots of Ligand Entry, Malleable Binding and Induced Helical Movement in P-Glycoprotein. *Biological Crystallography* **2015**, *71*, 732–741.
2. Le, C.A.; Harvey, D.S.; Aller, S.G. Structural Definition of Polyspecific Compensatory Ligand Recognition by P-Glycoprotein. *IUCrJ* **2020**, *7*, 663–672.
3. Barbieri, A.; Thonghin, N.; Shafi, T.; Prince, S.M.; Collins, R.F.; Ford, R.C. Structure of ABCB1/P-Glycoprotein in the Presence of the CFTR Potentiator Ivacaftor. *Membranes (Basel)* **2021**, *11*, 923.
4. Nosol, K.; Romane, K.; Irobalieva, R.N.; Alam, A.; Kowal, J.; Fujita, N.; Locher, K.P. Cryo-EM Structures Reveal Distinct Mechanisms of Inhibition of the Human Multidrug Transporter ABCB1. *Proceedings of the National Academy of Sciences* **2020**, *117*, 26245–26253.
5. Hamaguchi-Suzuki, N.; Adachi, N.; Moriya, T.; Yasuda, S.; Kawasaki, M.; Suzuki, K.; Ogasawara, S.; Anzai, N.; Senda, T.; Murata, T. Cryo-EM Structure of P-Glycoprotein Bound to Triple Elacridar Inhibitor Molecules. *Biochem Biophys Res Commun* **2024**, *709*, 149855.
6. Consortium, U. UniProt: A Hub for Protein Information. *Nucleic Acids Res* **2015**, *43*, D204–D212.
